# The Role of Mental Imagery in Parkinson’s Disease Rehabilitation

**DOI:** 10.3390/brainsci11020185

**Published:** 2021-02-02

**Authors:** Amit Abraham, Ryan P. Duncan, Gammon M. Earhart

**Affiliations:** 1 Department of Physical Therapy, Faculty of Health Sciences, Ariel University, Ariel 4077625, Israel; 2 Navigation and Accessibility Research Center of Ariel University (NARCA), Ariel University, Ariel 4077625, Israel; 3 Program in Physical Therapy, Washington University in St. Louis School of Medicine, St. Louis, MO 63108, USA; duncanr@wustl.edu (R.P.D.); earhartg@wustl.edu (G.M.E.); 4 Department of Neurology, Washington University in St. Louis School of Medicine, St. Louis, MO 63110, USA; 5 Department of Neuroscience, Washington University in St. Louis School of Medicine, St. Louis, MO 63110, USA

**Keywords:** Parkinson’s disease, rehabilitation, mental imagery, motor, sensorimotor, sensory, dynamic neuro-cognitive imagery, motor imagery

## Abstract

Parkinson’s disease (PD) is a disabling neurodegenerative disease whose manifestations span motor, sensorimotor, and sensory domains. While current therapies for PD include pharmacological, invasive, and physical interventions, there is a constant need for developing additional approaches for optimizing rehabilitation gains. Mental imagery is an emerging field in neurorehabilitation and has the potential to serve as an adjunct therapy to enhance patient function. Yet, the literature on this topic is sparse. The current paper reviews the motor, sensorimotor, and sensory domains impacted by PD using gait, balance, and pain as examples, respectively. Then, mental imagery and its potential for PD motor and non-motor rehabilitation is discussed, with an emphasis on its suitability for addressing gait, balance, and pain deficits in people with PD. Lastly, future research directions are suggested.

## 1. Introduction

Whether called daydreaming, imagining, or fantasizing, creating and using mental images―known as mental imagery―is ubiquitous in many people’s day-to-day activities [1]. This cognitive process of mentally imaging motor acts, sensations, or sights, be them realistic or imaginary, has been shown to positively affect (and even re-shape) the body and mind and to result in improved quality and functionality of both [1,2]. Two mental imagery subtypes are motor imagery and dynamic neuro-cognitive imagery, both discussed in detail below. One population who may benefit greatly from mental imagery as a rehabilitative approach is people with Parkinson’s disease (PwP).

Parkinson’s disease (PD) impacts more than 6 million people worldwide and its prevalence is quickly increasing [3]. While clinical diagnosis of PD is based upon the presence of bradykinesia and other cardinal motor features, PD is more than just a motor disorder [4]. In fact, one can identify features of PD that range from motor to sensory. Treatments for PD are often aimed at controlling motor and non-motor manifestations using pharmacological and surgical means, which are only partially effective. Rehabilitation and exercise complement these treatments [5]. The purpose of this paper is to highlight the role of mental imagery as one rehabilitative approach and detail its unique qualities that potentially allow it to explicitly and specifically address PD manifestations that span from motor to sensorimotor to sensory. Given their high prevalence and clear impact on function and quality of life, we specifically highlight gait, balance and pain, arguing that each of these may be effectively addressed using mental imagery.

## 2. Gait as an Example of Motor Dysfunction in PD

Gait impairment may present relatively early in the course of PD and is viewed as a red flag for emerging disability [6]. Early changes in gait include: reductions in arm swing, gait speed and step length; increases in interlimb asymmetry [7] and variability; and losses of gait automaticity [7] reflected in difficulty performing complex gait tasks such as turning or dual tasking [7]. With disease progression, gait slows further with shuffling steps, increased cadence, and more time spent in double limb support. Automaticity also continues to deteriorate, as problems emerge with gait initiation and turning is more disrupted. PwP may also begin to experience festination and freezing of gait (FOG). In late stages of the disease, as gait continues to worsen, FOG may become more prevalent and individuals may rely on assistive devices or eventually utilize a wheelchair for mobility [8].

The mechanisms underlying gait dysfunction in PD are not fully understood, and many different brain regions with the cerebral cortex, subcortical areas and cerebellum have been implicated. Imaging studies have shown changes in neural activity, connectivity and tissue volume. A recent meta-analysis of gait neuroimaging studies in PD concluded that decreased activation of the supplementary motor area along with activation of the cerebellar locomotor region are consistent findings [9]. However, firm conclusions regarding mechanisms of gait dysfunction are difficult given the variety of study methods, the relative lack of studies relating specific gait features to specific neural changes, and the paucity of prospective longitudinal studies that track changes in neuroimaging findings along with changes in gait. What is clear is that gait disorders are complex and multifactorial. Given this complex, multifactorial nature of gait disorders in PD, a multimodal approach to treatment is advisable [10].

Commonly recommended treatment approaches include dopaminergic and cholinergic medications, neurostimulation, physical activity and exercise, physical therapy, cueing and cognitive/behavioral therapy [10]. Mental imagery falls within this category of cognitive/behavioral therapy and is an approach that merits greater attention as a means of addressing gait difficulties in PD.

## 3. Postural Instability as an Example of Sensorimotor Dysfunction in PD

Sensorimotor deficits in PD [11] include, among others, impaired proprioception [12,13], disrupted sensitivity to motion [14], and altered awareness of limb position (i.e., kinesthesia) [15] and bodily orientation [16]. Although often unobserved, such deficits precede the onset of the motor [17] signs in approximately 20% of patients [18]. Such deficits can directly impact afferent sensory inputs and/or their processing in the central nervous system [19] as well as affect proper use of internal, proprioceptive stimuli and feedback [11,14].

Sensorimotor dysfunction contributes to postural stability and balance control deficits common among PwP [20]. A study with 109 participants noted that 68.3% of PwP experienced a single fall and 50.5% experienced at least two falls over a one-year period [21]. Falls may result in physical injury and in psychological complications, such as fear of falling, which itself could further compromise balance and postural control [22]. Typical characteristics of balance deficits in PD include diminished sway, reduced base of support, rigidity, abnormal inter-segmental coordination, and postural malalignment [20,23,24,25,26,27]. Related somatosensory deficits in PwP include problems with orientation to sensory and somatosensory information as well as processing issues [25,28]. Such deficits negatively impact sensorimotor integration [20] thus degrading proprioception, joint position sense, and tactile stimulus estimation [29,30,31], all of which are involved in balance control. In PD, such sensorimotor deficits interrelate with cognitive concerns, such as challenges with attention and temporal orientation [32].

Considered as a whole, this range of deficits faced by PwP seems to negatively impact the use of somatosensory information [20,24,25,26] for selecting a proper postural motor strategy [33], including switching between strategies [34,35]. The observed over-reliance on visual information seen in PwP [36] for controlling posture [37] could, therefore, be a compensatory strategy to overcome deficits in the somatosensory [38] and cognitive realms [30].

Current pharmacologic (e.g., levodopa) and invasive (e.g., deep brain stimulation) therapies do not adequately address sensorimotor and balance deficits in PwP [39,40]. Multiple non-pharmaceutical and non-invasive therapies, including physical therapy [41], dance [42], Tai Chi [43], and music-based movement therapy [44] have all shown beneficial effects on balance in PwP. However, the literature on this topic is inconclusive due to a lack of studies with long-term follow-up [45] and gaps in synthesis of research findings across the spectrum of PD disability [46]. Aligning with previous literature [47], these gaps include a lack of studies to determine the impact of innovative adjacent therapies, such as mental imagery, as a means of addressing balance deficits in PwP.

## 4. Pain as an Example of Sensory Dysfunction in PD

Among the sensory impairments experienced by PwP is pain, defined by the International Association for the Study of Pain as an unpleasant sensory and emotional experience associated with, or resembling that associated with, actual or potential tissue damage [48]. More than 60% of PwP experience pain [49], which may be present at or prior to diagnosis [50,51]. Pain ranks as a highly bothersome symptom throughout the course of PD [52]. Despite the high prevalence and devastating impact of pain in PD, it remains an under-reported and under-treated non-motor symptom.

Though the pathophysiology of pain in PwP is not fully understood, there are certain structures and circuits implicated. PwP who report having pain may demonstrate a reduced threshold for pain compared to those without pain [53,54,55]. Studies suggest irregularities in both transmission and processing of pain signals at the levels of the spinal cord [56] and cortex [57], respectively. In PwP, the impact of dopaminergic deficiency on pain remains under investigation. Given that pain may increase in “OFF” periods of the medication cycle compared to on [58] and that pain may be reduced following deep brain stimulation [59], dopaminergic dysfunction likely plays a key role in pain in PwP.

There are multiple types of pain in PwP. Ford reports five classifications of pain in PD: (1) musculoskeletal, (2) radicular, (3) dystonic, (4) akathisia, and (5) central [60]. Musculoskeletal pain complaints are the most common among people with PD, with the low back as one of the most frequently reported sites of pain [61,62]. Given that low back pain (LBP) is highly prevalent and is negatively associated with self-reported physical activity [63] and quality of life in PD [61,63], we suggest LBP in PwP may be appropriately targeted with mental imagery, especially when combined with movement-based therapy.

## 5. Distorted Body Schema as a Target for Rehabilitation Using Mental Imagery

Perceptual factors such as attentional focus, proprioception, awareness, and self-motion perception are all inter-related [17,64,65,66] and impact motor, sensorimotor, and sensory functions and the dynamic interaction between them [17,64,65,66,67,68] in PD [69,70,71,72,73,74,75,76]. One possible path through which these factors could impact motor and non-motor functions in PwP is via the body schema, which is the online mental representation of one’s body segments in space and their spatial relationships to each other [77]. Body schema has a vital role in perception and action [78], motor control [79], and mental imagery [80] (see below). Body schema is governed by a set of fronto-parietal brain networks which code for the online dynamic proprioceptive representation of the body [79,80], an essential factor for proper daily life functioning.

In PwP, body schema may be distorted [74,75,81] and may include inaccurate mental estimates of body part size [81,82] and location and position in space [83]. Body schema deficits could, in turn, affect motor planning and execution processes [84], thus promoting motor deficits (e.g., hypometric movements [12,19], postural malalignments [85,86]) and non-motor changes such as increased nociceptive sensitivity [87], altered nociceptive integration [88], and altered mental imagery abilities [89,90].

The perception–motor pathway is thought to be bidirectional [17,89,91] and seems to align with motor learning and control theories in people with and without PD [19,92]. Among PwP, for example, reduced volume and quality of movement (e.g., hypometric movements, diminished movement repertoire, lack of physical activity) could reduce peripheral kinesthetic (and other) afferent stimuli [93], thus potentially impeding body schema, given the importance of such information flow to body schema formation and maintenance [94].

The full spectrum of associations between perceptual and motor deficits, and especially the role of the former on PD symptoms and rehabilitation, is yet to be fully revealed [89]. Current PD therapies―pharmaceutical, invasive, and physical alike―are limited in their ability to alleviate motor (gait, balance) and non-motor symptoms [95]. Simultaneously, therapies that promote patients’ cognitive and motor independence and self-potency via providing them with practical explicit tools and strategies that can be consciously used in daily life scenarios are limited. Specifically, non-pharmaceutical and non-invasive therapies that explicitly address body schema deficits and their reframing in PwP are sparse. Therefore, development of therapeutic approaches that can be specifically tailored to PwP and provide a structured framework for patient-based stimuli and training [96] to include cognitive–perceptual components (e.g., attention, proprioception, awareness, self-motion perception) is essential [17,97,98,99,100,101]. One such potential approach is mental imagery, which has the potential to address distortions in body schema.

## 6. Mental Imagery: Background

Mental imagery is a term used to describe the cognitive process of simulating sensations, actions, or other types of experiences [102] through generating and using mental images, including metaphors. Mental imagery can be performed in the absence of appropriate sensory input [103,104] or while the imaged stimulus is available to the imager [105,106,107]. When related to movement, mental imagery can be done with or without physical execution of the imaged movement [108,109] as well as while observing the movement performed by another individual or a video (aka “action observation”) [110,111]. A strong linear correlation exists between overt physical execution and mental imagery [112,113], spanning temporal (i.e., similarity in time to complete actual and imaged tasks) and spatial (i.e., activated neural pathways and brain regions) spheres [104,114,115]. Such similarities give rise to sensory and motor experiences and effects that are associated with both mental imagery and perception [68,116] and could explain mental-imagery-related effects on peripheral and central neural events, including in PwP [117]. Therefore, mental imagery appears to have potential as a means of approaching novel and learned motor tasks alike [118] and can be used for motor planning, execution, and control purposes, including performance enhancement. Among the advantages of mental imagery as a rehabilitation method are no risk of physical injury, independence of level of motor capability, high availability/accessibility, low financial costs, and no need for equipment. Furthermore, mental imagery can explicitly and precisely target various motor (e.g., range-of-motion [119]) and non-motor (i.e., sensory and cognitive) aspects of performance, including pain [119], motivation, and self-confidence [120]. Specifically relevant for PD, mental imagery can be used even when physical mobility is limited, such as in advanced stages of the disease. Mental imagery offers PwP and therapists a wide range of delivery possibilities: individually or in a group, and physically or remotely/virtually. These options make mental imagery highly relevant for various PD communities, including remote and under-served ones, thus addressing gaps and future directions identified by the previous literature [121,122,123].

Mental imagery’s mechanisms of effect are not fully understood and include both psychological and physiological ones [103,115,124]. Suggested psychological mechanisms of effect include facilitating cognitive elements regarding the skill (i.e., learning what to do), such as breaking down the skill into its components [125], attentional focus [124], (Gose and Abraham, under review), and different execution patterns to promote learning of movement strategies [92,115]. Suggested physiological mechanisms of effect include neural changes in the central nervous system, resulting in greater relaxation and altered programming of the motor system [126].

Given that: (1) perception relies on motor action [91,127]; and (2) motor functioning is impacted by somatosensory information [17], mental imagery could serve as a promising rehabilitative approach toward improving both perceptual–cognitive and motor functioning. This is based on mental imagery’s engagement of neural circuits that overlap with overt motor execution [124,128]; and on MI’s reliance on and usage of sensory and somatosensory (i.e., kinesthetic and proprioceptive) information [128,129]. Further, prior studies [107,130] and evidence obtained from a focus group of PwP indicate that the self-selected use of mental imagery in everyday activities [131] support the benefits that may stem from integrating mental imagery in PD rehabilitation.

Two subtypes of mental imagery are motor imagery practice (MIP) and dynamic neuro-cognitive imagery (DNI). MIP, which consists of the mental rehearsal of a motor act without overt physical execution, is the most widely used and researched approach to mental imagery [115,124,132]. Less well known and less studied is DNI, a systematized mental imagery method for motor and cognitive retraining which was adapted from “the Franklin Method” [133,134]. DNI utilizes a variety of mental imagery categories (e.g., emotional, anatomical, biomechanical, metaphorical), modalities (e.g., visual, kinesthetic, auditory) and mental-imagery-related assistive tools (e.g., self-touch [135] and self-talk [136]). DNI combines mental imagery (including MIP) with actual movement execution within various motor contexts, ranging from basic activities of daily living (e.g., standing up from a chair, lifting arms) to more advanced functions (e.g., single-leg balance, turning) [130]. The DNI pedagogical process introduces participants to the concept of mental imagery, its advantages and ease of use, and teaches them various ways to utilize it along with motor performance during functional tasks (e.g., sitting down, standing up, walking and turning). In doing so, DNI addresses various cognitive aspects associated with motor planning and performance, such as efficiency, proprioception, body schema, attentional focus, and dual tasking. The beneficial effects of DNI on motor and non-motor functions have been recently demonstrated in dancers and PwP [106,107,130]. Given that DNI, unlike MIP, has been empirically studied only in recent years and is less known by both clinicians and researchers, the current paper focuses on introducing its qualities that may be specifically relevant for PD rehabilitation and that should be further investigated.

## 7. The Suitability of Mental Imagery for PD Rehabilitation

Mental imagery is a recommended method for neurorehabilitation [137,138] and is especially promising for PD rehabilitation [99,139,140,141] as supported by: (1) its core role in motor, sensorimotor, and cognitive functioning [142,143,144], and (2) its ability to reproduce [145] and even potentially enhance [105] availability and quality of afferent sensory information from the body, including specific tissues and body parts [105]. Care should be taken to ensure that individuals with cognitive symptoms receive an appropriate neuro-psychiatric assessment to verify that they may benefit from a mental imagery approach. For those with adequate cognitive capacity, mental imagery may play a role in sensory re-weighting processes [146,147] relevant for gait, balance, and pain. The following sections specifically review evidence to date regarding the use mental imagery in each of these areas.

## 8. Mental Imagery to Address Gait in PD

PwP maintain the ability to image walking tasks as evidenced by performance on the Gait Imagery Questionnaire [148], which assesses visual and kinesthetic motor imagery. The ability to mentally image gait vividly and accurately is similar in PwP and controls and does not seem to correlate with actual walking performance, though PwP may perform mental imagery tasks more slowly than controls [90]. Mental imagery speed and vividness may be enhanced through use of visual cues [149] and potentially through using action observation [110,111]. The preserved ability for mental imagery and use of cueing suggests that even those with poor walking performance may still be able to image walking effectively and therefore potentially benefit from mental imagery as a strategy to compensate for gait impairments [150]. However, patterns of brain activation during imaged walking differ between controls and PwP. For example, those with PD have reduced activity in globus pallidus and increased activity in the supplementary motor area during imaged gait, particularly for complex tasks like imaged turning [151] and imaged backward walking [152]. Both reports did not provide details regarding the type of imagery used. Of note, the investigation of imaged visual cues (i.e., using the mental image of the visual cue without it being perceptually available) and its potential benefits on gait in PwP is at its infancy [107,130].

Among PwP with FOG, there is evidence of alterations in motor imagery that are not noted in those without FOG. Neuroimaging studies suggest that during imaged walking, those with FOG have greater activity in the mesencephalic locomotor region than those without freezing of gait, and this hyperactivity in MLR correlates with severity and duration of FOG [153]. Those with FOG have also been noted to have reduced activity in the globus pallidus during mentally imaged gait [154]. Perceptual motor studies demonstrate a mismatch between imaged and actual walking times when passing through doorways [155], a problem that may be uniquely associated with FOG. It remains to be seen whether training in mental imagery could facilitate different brain activation patterns that rely less on the MLR and/or a better match between mentally imagined and actual walking times and be used as a means of addressing FOG.

Relatively few studies have directly asked whether mental imagery training can improve walking performance in PwP. We think that the area holds much promise and emerging evidence supports the use of DNI for this purpose [107,130]. DNI not only provides the combination of mental imagery and movement, it also provides participants with mental imagery-based cues which are based on scientific information (e.g., anatomy, biomechanics, motor control) that may help individuals with mental imagery use and retrieval when necessary in their daily life functioning which includes gait tasks [149]. Specifically, the DNI process allows for the conversion of externally-generated cues, known to be effective for PwP [156,157], into internally-generated ones. Internally-generated cues are readily accessible, enhance autonomy and self-empowerment, and are known to be effective for improving walking [71]. Furthermore, DNI delivered over multiple sessions across multiple weeks improved mental imagery abilities as well as motor and spatial cognitive functions relevant to gait [130]. Similarly, a 12-week program of combined visual–kinesthetic motor imagery practice combined with physical practice proved superior to a physical practice only condition for improving bradykinesia during the Timed Up and Go task [141]. However, a single session of MIP with a kinesthetic emphasis did not result in effects on gait [158]. Another study found that visual MIP was no different from relaxation in terms of effects on gait in a 6-week intervention [159]. Clearly, the verdict is still out as to the best mental imagery approach (including content, modality, perspective, etc.) for use in PD rehabilitation and additional evidence is needed. Related to this, a recent survey showed that only 60% of healthcare professionals have an awareness of mental imagery as a strategy to address gait impairments in PD, and only 45% of them actually apply mental imagery within their practice [160].

## 9. Mental Imagery to Address Balance in PD

Mental imagery could be an advantageous method for balance retraining in PwP as it can explicitly address specific psychological (e.g., self-confidence, attentional focus, and self-efficacy) [161] and motor (e.g., center of mass, base of support, and central axis) [162,163] determinants associated with balance. Mental imagery’s specificity was further demonstrated via resultant brain activity which corresponded with the varying levels of difficulty of an imaged balance task [143]. This sensitivity of mental imagery [139] adds to its potential for specificity in balance retraining, a component previously recommended in PD rehabilitation [97].

Studies assessing the effect of mental imagery training on balance measures in PwP are limited. In a study assessing the effect of group treatment of combined MIP–physical therapy versus physical therapy only, positive trends toward improvements in balance were noted in the combined group [141]. In a case-study of a single participant with PD, a 3-month neurocognitive rehabilitation program involving mental imagery which included 20 sessions (one hour each, twice per week; no further details regarding the type of imagery were provided) resulted in improvements in balance and reduction of risk of falls during both “OFF” and “ON” phases, as measured with the Tinetti Balance and Gait Evaluation Scale [164]. In another study, the effect of a 2-week DNI compared to reading and exercise interventions on balance (using self-reported questionnaires) and balance confidence (using the Activities-Specific Balance Confidence Scale) in individuals with mild–moderate PD [130] was examined and no significant differences between groups were noted following the intervention. However, participants in the DNI group reported self-perceived improvements in balance following the intervention [130].

## 10. Mental Imagery to Address Pain in PD

People with pain, who do not have PD, have reported vivid mental images associated with their pain [165]. Additionally, the mental images of pain were associated with anxiety, depression, and catastrophizing [165]. Mental imagery training has the potential to influence the sensory and emotional experience in people with pain. Volz and colleagues suggest that mental imagery may alter motor cortex activity resulting in pain modulation [166].

Studies using mental imagery as a means for reducing pain in PwP are limited. In one study, investigators studied the effect of DNI on mental imagery ability, PD severity, and motor and non-motor function in PwP [130]. While participants in this study demonstrated improvements in mental imagery ability as well as motor and cognitive functions, pain, as measured by the Brief Pain Inventory, was unchanged [130]. However, the DNI intervention did not specifically address pain or pain-related aspects. There is also a case report in which a participant with PD completed 20 sessions of neurocognitive rehabilitation with motor imagery [164]. In this intervention, the participant performed motor imagery of functional movements (e.g., sit to stand, walking) prior to physical performance of these tasks. There is no specific mention of mental imagery components to target pain. The participant reported, via the Visual Analog Scale, a 5.3-point reduction in pain following the intervention. Pain was further reduced at the 3-month follow-up visit. It is important to note that the authors state the lower limb pain in the participant with PD was a freezing prodrome, which may not be representative of the musculoskeletal pain experienced in PwP.

In PwP with chronic pain, the description of their pain experience may generate mental images. Specifically, PwP may report a distorted body schema [89]. This may extend to PwP who have LBP. Abraham and colleagues used self-drawn pelvic drawings to demonstrate that people with PD have the ability to change their misperceived body schema following an intensive 2-week DNI training [107]. However, whether there is a relationship between distorted body schema and pain and whether pain changes in response to a change in perceived body schema is unclear in PwP. Further, PwP with LBP report that LBP impacts their ability to perform activities like standing, lifting, walking, and sleeping [63]. It is unclear whether pain during these activities causes PwP with LBP to generate mental images associated with that pain. Better understanding of physical, sensory, and emotional aspects of the PwP in pain could aid the development of customized mental imagery programs targeted at reducing pain. Much work remains to be done to determine the type and content of mental imagery that is most effective for pain reduction in PwP. Given that there are multiple types of pain in PwP, future work should determine which forms of mental imagery are most effective for different pain syndromes in PwP. Further, investigators should seek to understand how mental imagery influences the known neurophysiologic mechanisms of pain in PwP. Despite the lack of evidence supporting the efficacy of mental imagery to reduce pain in PD, further investigation is warranted because it appears to be safe, low risk, and highly accessible for both PwP and therapists.

## 11. Summary

Mental imagery’s mechanisms of effect involve multisensory processing and somatosensory integration [17,76,167,168]. As such, mental imagery may be an appropriate therapeutic approach capable of addressing the wide array of PD-related motor and non-motor symptoms via numerous cognitive channels, such as attentional focus and body schema. This is especially relevant given that mental imagery ability is well-preserved in PwP compared to older adults [90,169], although it exhibits somewhat different characteristics [90,151]. Mental imagery has been attracting increasing scientific and clinical investigation in recent years. Yet, major gaps in knowledge exist thus limiting its substantial integration within PD rehabilitation. Given the limited literature, best mental imagery practices in PD rehabilitation, including elements such as specific contents, dosage, and mode of delivery (e.g., individual versus group sessions and face-to-face versus remote training) are yet to be revealed. Further, tailoring mental imagery protocols to PD subtypes (e.g., with or without FOG, with and without cognitive impairment) has yet to be explored. Testing the suitability and short- and long-term effectiveness of different mental imagery approaches (e.g., MIP, DNI) and characteristics (e.g., modality, perspective) in subgroups of PwP, and to address specific motor, sensorimotor, and sensory concerns, are important steps on the path forward if we are to utilize mental imagery to the greatest possible advantage.
